# Supplementary material for: Women’s perceptions and experiences of reproductive coercion and abuse: a qualitative evidence synthesis
Source: PLoS One. 2021 Dec 21;16(12):e0261551. doi: 10.1371/journal.pone.0261551 (PMC8691598; doi:10.1371/journal.pone.0261551)
Supplement: S1 Table — Completed ENTREQ statement for enhancing transparency in reporting the synthesis of qualitative research. (DOCX) [file pone.0261551.s002.docx]

**Women’s perceptions and experiences of reproductive coercion and abuse: a qualitative evidence synthesis**

Jessica E. Moulton^1*^, Martha Isela Vazquez Corona^1^, Cathy Vaughan^1^, Meghan A. Bohren^1^

* Corresponding author: Jessica Moulton, jessica.moulton@monash.edu

^1^Gender and Women’s Health Unit, Centre for Health Equity, School of Population and Global Health, University of Melbourne, Carlton, VIC, Australia

**Authors’ email & ORCID ID**

Jessica E. Moulton:

- [jessica.moulton@monash.edu](mailto:jessica.moulton@monash.edu)
- 0000-0001-7172-9470

Martha Isela Vazquez Corona:

- [martha.vazquezcorona@unimelb.edu.au](mailto:martha.vazquezcorona@unimelb.edu.au)

Cathy Vaughan:

- cmvaug@unimelb.edu.au
- 0000-0003-3988-8222

Meghan A. Bohren:

- [Meghan.bohren@unimelb.edu.au](mailto:Meghan.bohren@unimelb.edu.au)
- 0000-0002-4179-4682

**S1 Table. ENTREQ Statement. Completed ENTREQ statement for enhancing transparency in reporting the synthesis of qualitative research.**

| **Enhancing transparency in reporting the synthesis of qualitative research: the ENTREQ statement** | | | |
| --- | --- | --- | --- |
| **No** | **Item** | **Guide and description** | **Page#** |
| **1** | Aim | To synthesise the existing literature on the perceptions and experiences of women who have encountered reproductive coercion | 4 |
| **2** | Synthesis methodology | Thematic synthesis | 9 |
| **3** | Approach to searching | Pre-planned comprehensive search strategies to seek all available studies | 8 |
| **4** | Inclusion criteria | Qualitative research methods (data collection and analysis).  Participants: Women who have encountered reproductive coercion (either defined by themselves or the researcher).  Topic: Experiences and perceptions of women who have encountered reproductive coercion  Type of publication: Primary Data Collection. No language or year limits | 7-8 |
| **5** | Data sources | EMBASE, MEDLINE, CINAHL to identify literature using qualitative methods (focus groups or interviews) to explore women’s perceptions and experiences of reproductive coercion  Search was last updated on 20^th^ June 2019 | 8 |
| **6** | Electronic Search strategy | Literature search terms are described in detail in “Appendix 2: Search Strategies” | 8 |
| **7** | Study screening methods | The titles and abstracts of retrieved citations were scanned by three reviewers. Full papers were accessed for all potentially relevant abstracts. Full papers were reviewed by one reviewer and were included if they met the inclusion criteria | 8 |
| **8** | Study characteristics | The characteristics of the included studies are presented in Appendix 1. | Appendices page 11-15 |
| **9** | Study selection results | The studies screened are described in Figure 1 (flow diagram) | 12 |
| **10** | Rationale for appraisal | One reviewer formally assessed quality of findings | 9 |
| **11** | Appraisal items | The CASP tool was used to appraise all included studies | 9 |
| **12** | Appraisal process | Quality assessment was conducted by one reviewer | 9 |
| **13** | Appraisal results | Study appraisal results are included in Appendix 3: Characteristics of Included Studies. | Appendices page 11-15 |
| **14** | Data extraction | One reviewer conducted data extraction using an adapted template. All text under Methods and Results were considered data from the primary studies. Data was extracted on an electronic template and then entered into a computer software for data management and analysis. | 9 |
| **15** | Software | NVivo 12.0 | 10 |
| **16** | Number of reviewers | Three reviewers for abstract/title screening, One reviewer for remainder of review process. | 8-9 |
| **17** | Coding | Line by line coding was conducted by one reviewer | 9-10 |
| **18** | Study comparison | 5 studies were selected initially to develop a code book. Subsequent studies were then coded using the code book, with new concepts created where necessary | 9-10 |
| **19** | Derivation of themes | Themes were derived inductively | 9 |
| **20** | Quotations | Quotations from the primary studies are provided throughout the results | 14-27 |
| **21** | Synthesis output | The key concepts demonstrate key experiences and perspectives of women in existing qualitative work, across society, and will aid in the development of interventions as well as guiding further research. | 27- 33 |
