# Supplementary material for: Women’s perceptions and experiences of reproductive coercion and abuse: a qualitative evidence synthesis
Source: PLoS One. 2021 Dec 21;16(12):e0261551. doi: 10.1371/journal.pone.0261551 (PMC8691598; doi:10.1371/journal.pone.0261551)
Supplement: S2 Table — Search strategies for MEDLINE, Embase, and CINAHL databases. (DOCX) [file pone.0261551.s003.docx]

**Women’s perceptions and experiences of reproductive coercion and abuse: a qualitative evidence synthesis**

Jessica E. Moulton^1*^, Martha Isela Vazquez Corona^1^, Cathy Vaughan^1^, Meghan A. Bohren^1^

* Corresponding author: Jessica Moulton, jessica.moulton@monash.edu

^1^Gender and Women’s Health Unit, Centre for Health Equity, School of Population and Global Health, University of Melbourne, Carlton, VIC, Australia

**Authors’ email & ORCID ID**

Jessica E. Moulton:

- [jessica.moulton@monash.edu](mailto:jessica.moulton@monash.edu)
- 0000-0001-7172-9470

Martha Isela Vazquez Corona:

- [martha.vazquezcorona@unimelb.edu.au](mailto:martha.vazquezcorona@unimelb.edu.au)

Cathy Vaughan:

- cmvaug@unimelb.edu.au
- 0000-0003-3988-8222

Meghan A. Bohren:

- [Meghan.bohren@unimelb.edu.au](mailto:Meghan.bohren@unimelb.edu.au)
- 0000-0002-4179-4682

**S2 Table. Search Strategies.**

Search strategies for MEDLINE, Embase, and CINAHL databases.

**MEDLINE: Epub Ahead of Print, In-Process & Other Non-Indexed Citations, MEDLINE Daily and MEDLINE 1946 to 20 June 2019, Ovid**

| **#** | **Searches** | **Results** |
| --- | --- | --- |
| 1 | gender-based violence/or intimate partner violence/or spouse abuse/ | 8864 |
| 2 | reproduct* coercion.mp. [mp=title, abstract, original title, name of substance word, subject heading word, floating sub-heading word, keyword heading word, organism supplementary concept word, protocol supplementary concept word, rare disease supplementary concept word, unique identifier, synonyms] | 76 |
| 3 | reproduct* control.mp. [mp=title, abstract, original title, name of substance word, subject heading word, floating sub-heading word, keyword heading word, organism supplementary concept word, protocol supplementary concept word, rare disease supplementary concept word, unique identifier, synonyms] | 1663 |
| 4 | pregnan* coerc*.mp. [mp=title, abstract, original title, name of substance word, subject heading word, floating sub-heading word, keyword heading word, organism supplementary concept word, protocol supplementary concept word, rare disease supplementary concept word, unique identifier, synonyms] | 22 |
| 5 | contracept* sabotage.mp. [mp=title, abstract, original title, name of substance word, subject heading word, floating sub-heading word, keyword heading word, organism supplementary concept word, protocol supplementary concept word, rare disease supplementary concept word, unique identifier, synonyms] | 8 |
| 6 | reproduct* abuse.mp. [mp=title, abstract, original title, name of substance word, subject heading word, floating sub-heading word, keyword heading word, organism supplementary concept word, protocol supplementary concept word, rare disease supplementary concept word, unique identifier, synonyms] | 2 |
| 7 | (reproduct* adj10 coerc*).mp. [mp=title, abstract, original title, name of substance word, subject heading word, floating sub-heading word, keyword heading word, organism supplementary concept word, protocol supplementary concept word, rare disease supplementary concept word, unique identifier, synonyms] | 160 |
| 8 | pregnan*.mp. [mp=title, abstract, original title, name of substance word, subject heading word, floating sub-heading word, keyword heading word, organism supplementary concept word, protocol supplementary concept word, rare disease supplementary concept word, unique identifier, synonyms] | 965263 |
| 9 | reproduct*.mp. [mp=title, abstract, original title, name of substance word, subject heading word, floating sub-heading word, keyword heading word, organism supplementary concept word, protocol supplementary concept word, rare disease supplementary concept word, unique identifier, synonyms] | 278117 |
| 10 | intimate partner violence.mp. [mp=title, abstract, original title, name of substance word, subject heading word, floating sub-heading word, keyword heading word, organism supplementary concept word, protocol supplementary concept word, rare disease supplementary concept word, unique identifier, synonyms] | 7288 |
| 11 | gender-based violence.mp. [mp=title, abstract, original title, name of substance word, subject heading word, floating sub-heading word, keyword heading word, organism supplementary concept word, protocol supplementary concept word, rare disease supplementary concept word, unique identifier, synonyms] | 831 |
| 12 | spouse abuse.mp. [mp=title, abstract, original title, name of substance word, subject heading word, floating sub-heading word, keyword heading word, organism supplementary concept word, protocol supplementary concept word, rare disease supplementary concept word, unique identifier, synonyms] | 7348 |
| 13 | domestic violence.mp. [mp=title, abstract, original title, name of substance word, subject heading word, floating sub-heading word, keyword heading word, organism supplementary concept word, protocol supplementary concept word, rare disease supplementary concept word, unique identifier, synonyms] | 9872 |
| 14 | 1 or 2 or 3 or 4 or 5 or 6 or 7 | 10643 |
| 15 | 8 or 9 | 1171821 |
| 16 | 10 or 11 or 12 or 13 | 18968 |
| 17 | 15 and 16 | 2866 |
| 18 | 14 or 17 | 12066 |
| 19 | Limit 18 to “qualitative (best balance of sensitivity and specificity)” | **4448** |

**Embase Ovid**

**Embase classic + Embase 1947 to 20 June 2019**

| **#** | **Searches** | **Results** |
| --- | --- | --- |
| 1 | gender-based violence/or intimate partner violence/or spouse abuse/ | 11572 |
| 2 | reproduct* coercion.mp. [mp=title, abstract, heading word, drug trade name, original title, device manufacturer, drug manufacturer, device trade name, keyword, floating subheading word, candidate term word] | 88 |
| 3 | reproduct* control.mp. [mp=title, abstract, heading word, drug trade name, original title, device manufacturer, drug manufacturer, device trade name, keyword, floating subheading word, candidate term word] | 259 |
| 4 | pregnan* coerc*.mp. [mp=title, abstract, heading word, drug trade name, original title, device manufacturer, drug manufacturer, device trade name, keyword, floating subheading word, candidate term word] | 28 |
| 5 | contracept* sabotage.mp. [mp=title, abstract, heading word, drug trade name, original title, device manufacturer, drug manufacturer, device trade name, keyword, floating subheading word, candidate term word] | 10 |
| 6 | reproduct* abuse.mp. [mp=title, abstract, heading word, drug trade name, original title, device manufacturer, drug manufacturer, device trade name, keyword, floating subheading word, candidate term word] | 1 |
| 7 | (reproduct* adj50 coerc*).mp. [mp=title, abstract, heading word, drug trade name, original title, device manufacturer, drug manufacturer, device trade name, keyword, floating subheading word, candidate term word] | 191 |
| 8 | pregnan*.mp. [mp=title, abstract, heading word, drug trade name, original title, device manufacturer, drug manufacturer, device trade name, keyword, floating subheading word, candidate term word] | 1044554 |
| 9 | reproduct*.mp. [mp=title, abstract, heading word, drug trade name, original title, device manufacturer, drug manufacturer, device trade name, keyword, floating subheading word, candidate term word] | 354174 |
| 10 | intimate partner violence.mp. [mp=title, abstract, heading word, drug trade name, original title, device manufacturer, drug manufacturer, device trade name, keyword, floating subheading word, candidate term word] | 7271 |
| 11 | gender-based violence.mp. [mp=title, abstract, heading word, drug trade name, original title, device manufacturer, drug manufacturer, device trade name, keyword, floating subheading word, candidate term word] | 1110 |
| 12 | spouse abuse.mp. [mp=title, abstract, heading word, drug trade name, original title, device manufacturer, drug manufacturer, device trade name, keyword, floating subheading word, candidate term word] | 419 |
| 13 | domestic violence.mp. [mp=title, abstract, heading word, drug trade name, original title, device manufacturer, drug manufacturer, device trade name, keyword, floating subheading word, candidate term word] | 11945 |
| 14 | 1 or 2 or 3 or 4 or 5 or 6 or 7 | 11937 |
| 15 | 8 or 9 | 1307338 |
| 16 | 10 or 11 or 12 or 13 | 18180 |
| 17 | 15 and 16 | 3004 |
| 18 | 14 or 17 | 13429 |
| 19 | Limit 18 to “qualitative (best balance of sensitivity and specificity)” | **4543** |

**Cinahl, EbscoHost**

**Cinahl 1981 to 20 June 2019, EbscoHost**

| **#** | **Query** | **Results** |
| --- | --- | --- |
| S19 | S14 OR S17 (Limiters – **Clinical Queries: Qualitative – Best Balance**) | **1,450** |
| S18 | S14 OR S17 | 16,707 |
| S17 | S15 AND S16 | 1,194 |
| S16 | S10 OR S11 OR S12 OR S13 | 10,655 |
| S15 | S8 OR S9 | 128,447 |
| S14 | S1 OR S2 OR S3 OR S4 OR S5 OR S6 OR S7 | 16,342 |
| S13 | TI domestic violence OR AB domestic violence | 5,072 |
| S12 | TI spouse abuse OR AB spouse abuse | 141 |
| S11 | TI gender based violence OR AB gender based violence | 484 |
| S10 | TI intimate partner violence OR AB intimate partner violence | 5,615 |
| S9 | TI reproduct* OR AB reproduct* | 25,249 |
| S8 | TI pregnan* OR AB pregnan* | 110,400 |
| S7 | TI reproduct* N10 coerc* OR AB reproduct* N10 coerc* | 102 |
| S6 | TI reproduct* abuse OR AB reproduct* abuse | 54 |
| S5 | TI contracept* sabotage OR AB contracept* sabotage | 6 |
| S4 | TI pregnan* coerc* OR AB pregnan* coerc* | 47 |
| S3 | TI reproduct* control OR AB reproduct* control | 321 |
| S2 | TI reproduct* coercion OR AB reproduct* coercion | 83 |
| S1 | (MH "Intimate Partner Violence") OR (MH "Domestic Violence") OR (MH "Gender-Based Violence") | 15,923 |
