# Supplementary material for: Women’s perceptions and experiences of reproductive coercion and abuse: a qualitative evidence synthesis
Source: PLoS One. 2021 Dec 21;16(12):e0261551. doi: 10.1371/journal.pone.0261551 (PMC8691598; doi:10.1371/journal.pone.0261551)
Supplement: S3 Table — Critical appraisal of included studies using the CASP tool. (DOCX) [file pone.0261551.s004.docx]

**Women’s perceptions and experiences of reproductive coercion and abuse: a qualitative evidence synthesis**

Jessica E. Moulton^1*^, Martha Isela Vazquez Corona^1^, Cathy Vaughan^1^, Meghan A. Bohren^1^

* Corresponding author: Jessica Moulton, jessica.moulton@monash.edu

^1^Gender and Women’s Health Unit, Centre for Health Equity, School of Population and Global Health, University of Melbourne, Carlton, VIC, Australia

**Authors’ email & ORCID ID**

Jessica E. Moulton:

- [jessica.moulton@monash.edu](mailto:jessica.moulton@monash.edu)
- 0000-0001-7172-9470

Martha Isela Vazquez Corona:

- [martha.vazquezcorona@unimelb.edu.au](mailto:martha.vazquezcorona@unimelb.edu.au)

Cathy Vaughan:

- cmvaug@unimelb.edu.au
- 0000-0003-3988-8222

Meghan A. Bohren:

- [Meghan.bohren@unimelb.edu.au](mailto:Meghan.bohren@unimelb.edu.au)
- 0000-0002-4179-4682

**S3 Table: Critical appraisal. Critical appraisal of included studies using the CASP tool except for overall assessment**

| **Authors** | **Was there a statement of the aims of the research? (YES, NO, PARTIAL, UNCLEAR)** | **Given the aim of the study, was a qualitative methodology appropriate? (YES, NO, PARTIAL, UNCLEAR)** | **Was the research design appropriate to address the aims of the research? (YES, NO, PARTIAL, UNCLEAR)** | **Was the recruitment strategy appropriate to the aims of the research? (YES, NO, PARTIAL, UNCLEAR)** | **Was the relationship between the researcher and participants adequately considered? (YES, NO, PARTIAL, UNCLEAR)** | **Have ethical issues been taken into consideration? (YES, NO, PARTIAL, UNCLEAR)** | **Was the data analysis sufficiently rigorous? (YES, NO, PARTIAL, UNCLEAR)** | **Were the findings supported by the evidence? (YES/NO/PARTIAL/UNCLEAR)** | **How valuable is the research?** |
| --- | --- | --- | --- | --- | --- | --- | --- | --- | --- |
| **Alhusen 2019** | Yes | Yes | Yes | Yes | No | Yes | Yes | Yes | very valuable |
| **Bagwell-Grey 2019** | Yes (clearly stated pg 6) | Yes (womens experiences of sexual violence in iitimate partner relationships) | Yes | Yes (purposive sampling at DV agency/out-patient counselling program) | Partial (experienced researcher but no reflexivity) | Yes (ethical approval and informed consent) | Yes | Yes | Very Valuable |
| **Baird 2016** | Yes (clearly stated in pg 2401) | Yes (interest in exploring intentions and experiences) | Yes (IDI) | Yes (purposive sampling at women's support agencies/refuges through posters) | Partial (brief professional description of data section but no reflexity section) | Yes (ethical approval and informed consent) | Yes (extensive explanation of experiential analysis) | Yes | Very Valuable |
| **Barber 2018** | yes | yes | yes | yes | no | no | unclear | yes | valuable |
| **Batista 2020** | Yes | Yes | Yes | Yes | No | Yes | Partial | Yes | valuable |
| **Borrero 2008** | Yes (clearly stated in pg 151) | Partial (aim to explore relationship between pregnancy intention and contraceptive use, quantitative methods could also have been used as author presented some results in quantitative form (i.e. 44% of participants)) | Yes (Semistructured interviews | Yes (purposive sampling at 7 repro health clinics) | Partial (mentions researcher experienced in diverse pop but no reflexivity section | Partial (Ethical approval but no consent specified - discusses participants names being omitted) | Yes (content analysis) | Yes | Very Valuable |
| **Boyce 2020** | Yes | Yes | Yes | Yes | Partial | Yes | Yes | Yes | very valuable |
| **Campbell 1995** | Yes (clearly stated pg 214) | Yes (intentions) | Yes | Yes (purposive sampling from womens shelter) | No (no reflexive statements) | Partial (Consent but no ethical approval mentioned) | Unclear (very brief description of thematic analysis used) | Yes | Valuable |
| **Coggins 2003** | Partial | Unclear (relationships?) | unclear of aims | partial (convenience sampling) | No (no reflexive statements) | yes (ethical approval and informed consent) | yes | partial (made big assumptions) | somewhat valuable (made big assumptions) |
| **Dasari 2016** | yes (pg 104) | yes (interested in womens experiences) | yes | yes | no (no reflexive statements) | partial (only consent no ethical approval) | yes | yes | Very Valuable |
| **Douglas 2011** | Yes (pg 341) | partial (interested in relationship) | yes | yes | no | Partial (pseudonyms for confidentiality - no info on consent or ethical approval) | unclear - no real description (could be on other study?) | yes - quotes - no major assumptions | somewhat valuable |
| **Edin 2013** | yes (pg 2) | yes (interest in womens narratives) | yes (concerns about recruitment as carried out by coordinators of the shelter (could be issues with extra inclusion/exclusion criteria)) | Yes (purpsive sampling) | No (no reflexive statements) | partial (Ethical approval but no consent specified - but WHO ethical research standards followed) | yes | yes | Very Valuable |
| **Feld 2019** | Yes | Yes | yes | yes | partial | yes | yes | yes | very valuable |
| **Grace 2020** | Yes | Yes | Yes | Yes | Partial | Yes | Yes | Yes | very valuable |
| **Griffiths 2013** | yes (pg 1) | yes (pregnancy intention) | yes | yes (purposive sampling) | yes | yes | yes | yes | Very Valuable |
| **Hathaway 2005** | yes (pg 42) | yes (interested in womens repro choices) | yes | partial (convenience sampling) | partial (descrbes interviewer as bilingual and latina and appropriate for the study but no reflexive statements) | yes | yes | yes | Very Valuable |
| **Holliday 2018** | yes (cleary stated pg 205) | yes (womens narratives) | yes | Yes (purposive sampling) | no | partial (Ethical approval but no consent specified (participants may have already consented in RCT)) | yes | yes | Very Valuable |
| **Levesque 2019** | yes (pg 6-7) | yes (womens acknowledgement and percetptoons) | yes | yes | no | yes | yes | yes | Very valuable |
| **Miller 2007** | yes (pg 360) | yes (social context) | yes | yes | partial (description of interviewers female trained in the subject) | yes | yes | yes | very valuable |
| **Mitchell 2020** | Yes | Yes | Yes | Yes | No | Yes | Partial | Yes | valuable |
| **Moore 2010** | partial (defining the different types of reproductive control perpetrated by men, examining the behaviors along a temporal continuum.) | partial (could have done quant) | yes | yes | no | yes | yes | yes | valuable |
| **Nayebi 2019** | Yes | Partial | Unclear | Yes | No | Yes | Yes | Yes | somewhat valuable |
| **Nikolajski 2015** | yes (pg 216) | yes | yes | yes | no | partial (ethical approval no consent | yes | yes | Very valuable |
| **O’Connor-Terry 2020** | Yes | Yes | Yes | Yes | Partial | Yes | Yes | Yes | very valuable |
| **Obare 2020** | Yes | Yes | Yes | Yes | Partial | Yes | Partial | Yes | very valuable |
| **Ontiri 2021** | Yes | Yes | Yes | Yes | Partial | Yes | Yes | Yes | very valuable |
| **Paterno 2018** | yes | yes | yes | yes | no | yes | yes | yes | very valuable |
| **Paul 2015** | yes (pg 312) | yes (womens decisions, social context) | yes | yes | Partial (pg 314 - had field diary with reflections - A field diary with reflections and experiences was kept and used in the interpretation and contextualization of data) | yes | yes | yes | Very Valuable |
| **Puri 2011** | yes (pg 117) | yes | yes | yes | No (no reflexive statements) | yes | yes | yes | Very Valuable |
| **Tarzia 2020** | Yes | Yes | Yes | Yes | No | Yes | Partial | Yes | Valuable |
| **Uysal 2020** | Yes | Yes | Yes | Yes | Partial | Yes | Yes | Yes | very valuable |
| **Wilson-Williams 2008** | yes (pg 1118) | partial (discusses perceptions of domestic violence and relationship with contraception - potentially quant element would be more effective for relationship) | no - should have done IDI rather than FGD due to shy participants | yes (purposive sampling) | no | No (Meeting of community consent conducted but no formal consent or ethical approval mentioned | partial - too brief | Yes | somewhat valuable |
| **Wood 2020** | Yes | Yes | yes | yes | partial | yes | yes | yes | very valuable |
