# Supplementary material for: Women’s perceptions and experiences of reproductive coercion and abuse: a qualitative evidence synthesis
Source: PLoS One. 2021 Dec 21;16(12):e0261551. doi: 10.1371/journal.pone.0261551 (PMC8691598; doi:10.1371/journal.pone.0261551)
Supplement: S5 Table — Qualitative codebook developed during analysis. (DOCX) [file pone.0261551.s006.docx]

**Women’s perceptions and experiences of reproductive coercion and abuse: a qualitative evidence synthesis**

Jessica E. Moulton^1*^, Martha Isela Vazquez Corona^1^, Cathy Vaughan^1^, Meghan A. Bohren^1^

* Corresponding author: Jessica Moulton, jessica.moulton@monash.edu

^1^Gender and Women’s Health Unit, Centre for Health Equity, School of Population and Global Health, University of Melbourne, Carlton, VIC, Australia

**Authors’ email & ORCID ID**

Jessica E. Moulton:

- [jessica.moulton@monash.edu](mailto:jessica.moulton@monash.edu)
- 0000-0001-7172-9470

Martha Isela Vazquez Corona:

- [martha.vazquezcorona@unimelb.edu.au](mailto:martha.vazquezcorona@unimelb.edu.au)

Cathy Vaughan:

- cmvaug@unimelb.edu.au
- 0000-0003-3988-8222

Meghan A. Bohren:

- [Meghan.bohren@unimelb.edu.au](mailto:Meghan.bohren@unimelb.edu.au)
- 0000-0002-4179-4682

**S5 Table Codebook.**

Qualitative codebook developed during analysis.

| **Code Book** | **Examples** |
| --- | --- |
| **Manifestations of Reproductive Coercion** | *Specific behaviours that constitute reproductive coercion* |
| 1. **Contraceptive control** |  |
| - 1. **Contraceptive refusal** | *Condom refusal, or refusal to allow oral contraceptive pill (OCP)* |
| - 1. **Contraceptive sabotage** | *Overt contraceptive control such as throwing away birth control pills, pulling contraception (nuvaring or IUD) out of woman, biting holes in condom, etc* |
| - 1. **Contraceptive deception** | *Non-consensual condom removal, lying about using condom, dissuading woman from using contraceptive pill by exaggerating side effects or emotional coercion such as accusing woman of cheating if she continues with OCP, etc* |
|  |  |
| 1. **Pregnancy pressure/coercion** |  |
| - 1. **Emotional coercion** | *Verbal and emotional pressure such as talking about how badly partner wants a baby, threatening to leave if she doesn’t get pregnant, if woman advises she isn’t ready partner says “don’t you want to be a part of me, don’t you want to be a part of me forever?”* |
| - 1. **Physical coercion** | *Forced sex or rape with intention to get woman pregnant, or forced sex with indifference to whether woman is protected from pregnancy* |
| - 1. **Failure to conceive** | *Violence or harassment from partner if woman does not conceive* |
|  |  |
| 1. **Control of pregnancy outcome** |  |
| - 1. **Termination** | *Partner threatening violence to cause miscarriage, threatening to leave unless woman has abortion, demanding/forcing woman has abortion* |
| - 1. **Pregnancy continuation** | *Tactics such as:*  *Controlling woman’s movements to stop her having a termination*  *Begging*  *Badgering “you can’t kill my baby”*  *Making promises to support baby*  *Making woman feel guilty*  *Threats “if you kill my baby, I will kill you”* |
|  |  |
| **Reasons for Reproductive Coercion** |  |
|  |  |
| 1. **Reasons for reproductive coercion** |  |
| - 1. **To trap woman** | *Partner spoke about wanting to impregnate her to “tie her to him forever”* |
| - 1. **Wanting a child (Ego?)** | *Taking pride in being a father, believing it will make him stronger, happier* |
| - 1. **Incarceration** | *Partner monitored ovulatory cycles and sabotaged contraception efforts before incarceration*  *Pregnancy to maintain emotional and economic security while in prison*  *Impending incarceration meant partner wanted pregnancy to lesson chances of woman leaving him as she would be seen as less desirable and would invest more in relationship* |
| - 1. **Role of women** | Gender norms to bear children, not wanting to disobey or fail to fulfil husbands expectations in terms of child bearing, husbands emphasised need to produce children, strict gender roles and expectations on women around sexuality/fertility |
|  |  |
| **Effect of reproductive coercion on women?** |  |
|  |  |
| 1. **Consequences of reproductive coercion** | *Fear of STIs, psychological effects, unintended pregnancy* |
| 1. **Reasons for women’s compliance** |  |
| - 1. **Fear of abandonment** | *Love for partner eclipsed RC behaviour, fear that partner would end relationship* |
| - 1. **Dependence on partner** | *e.g. Financial dependence on partner* |
| - 1. **Fear of partner/violence** | *Fear that partner will hurt her if she disobeys wishes* |
| 1. **Women’s responses to reproductive coercion** |  |
| - 1. **Reaction – Minimising** | *Downplaying incident, “it started off as a bit of a joke when he removed the condom half way through”*  *manipulation led women to doubt seriousness of incident* |
| - 1. **Reaction – Shame** | *Women felt shame and internal conflict for disobeying husband and taking contraception in secret, women doubted their right to confront partner, difficulty self-identifying as victim* |
| - 1. **Reaction – Blaming self** | *Assuming responsibility, i.e. drinking at time of incident clouding recollection “maybe I said he could take off the condom”* |
| - 1. **Reaction – Anger** | *Woman mad that she took precautions and man took off condom like ‘he had the right to decide for her’* |
| - 1. **Women’s awareness of reproductive coercion** | *Awareness and acknowledgement depends on type of violence perpetrated*  *Factors facilitating awareness include:*  *Acquiring knowledge about the issue*  *Having a friend describe RC behaviour*  *Finding a new partner that respects contra choices* |
| - 1. **Barriers to awareness** | *Non-acknowledgement due to:*  *Assuming responsibility*  *Downplaying incident*  *Being in violent relationship*  *Difficulty identifying as victim* |
| - 1. **Help seeking** | *Ways in which women sought help* |
|  |  |
| **Resistance to Reproductive Coercion** |  |
|  |  |
| 1. **Forms of resistance** |  |
| - 1. **Fighting back** | *Women pushed off partner after realising there was no condom* |
| - 1. **Ending relationship** | *Partner coercion allowed woman to recognise controlling nature of relationship and end it* |
| - 1. **Covert contraception** | *Using contraception without partners knowledge i.e. secret depo injections, telling partner OCP are vitamins* |
| 1. **Partner’s reaction to resistance** | *Men interpreted women’s protests as emotional rejection*  *Physical and verbal violence towards woman if partner finds out she is taking covert contraception* |
| **Societal manifestations** |  |
|  |  |
| 1. **Reproductive coercion by in-laws** | *(in particular populations) Pregnancy pressure by in-laws, pressure for more children, mother-in-law influences husbands contraceptive decisions, source of pressure to have son* |
| 1. **Sex selection/Preference for sons** | *(in particular populations) Pressure for sons, clear expectation to have male child, termination of female fetus* |
| 1. **Differences in experiences across populations** | *Experiences of African American women as opposed to Caucasian, migrant women in Canada, etc* |
| 1. **Motherhood vs career?** | *Women torn between motherhood and their careers/schooling and influence in pregnancy intention (not sure if relevant here or even relevant at results level)* |
