# Supplementary material for: Women’s perceptions and experiences of reproductive coercion and abuse: a qualitative evidence synthesis
Source: PLoS One. 2021 Dec 21;16(12):e0261551. doi: 10.1371/journal.pone.0261551 (PMC8691598; doi:10.1371/journal.pone.0261551)
Supplement: S1 File — (DOCX) [file pone.0261551.s007.docx]

**Women’s perceptions and experiences of reproductive coercion and abuse: a qualitative evidence synthesis**

Jessica E. Moulton^1*^, Martha Isela Vazquez Corona^1^, Cathy Vaughan^1^, Meghan A. Bohren^1^

* Corresponding author: Jessica Moulton, jessica.moulton@monash.edu

^1^Gender and Women’s Health Unit, Centre for Health Equity, School of Population and Global Health, University of Melbourne, Carlton, VIC, Australia

**Authors’ email & ORCID ID**

Jessica E. Moulton:

- [jessica.moulton@monash.edu](mailto:jessica.moulton@monash.edu)
- 0000-0001-7172-9470

Martha Isela Vazquez Corona:

- [martha.vazquezcorona@unimelb.edu.au](mailto:martha.vazquezcorona@unimelb.edu.au)

Cathy Vaughan:

- cmvaug@unimelb.edu.au
- 0000-0003-3988-8222

Meghan A. Bohren:

- [Meghan.bohren@unimelb.edu.au](mailto:Meghan.bohren@unimelb.edu.au)
- 0000-0002-4179-4682

**Appendices**

**Appendix 1: ENTREQ Statement**

| **Enhancing transparency in reporting the synthesis of qualitative research: the ENTREQ statement** | | | |
| --- | --- | --- | --- |
| **No** | **Item** | **Guide and description** | **Page#** |
| **1** | Aim | To synthesise the existing literature on the perceptions and experiences of women who have encountered reproductive coercion | 4 |
| **2** | Synthesis methodology | Thematic synthesis | 9 |
| **3** | Approach to searching | Pre-planned comprehensive search strategies to seek all available studies | 8 |
| **4** | Inclusion criteria | Qualitative research methods (data collection and analysis).  Participants: Women who have encountered reproductive coercion (either defined by themselves or the researcher).  Topic: Experiences and perceptions of women who have encountered reproductive coercion  Type of publication: Primary Data Collection. No language or year limits | 7-8 |
| **5** | Data sources | EMBASE, MEDLINE, CINAHL to identify literature using qualitative methods (focus groups or interviews) to explore women’s perceptions and experiences of reproductive coercion  Search was last updated on 20^th^ June 2019 | 8 |
| **6** | Electronic Search strategy | Literature search terms are described in detail in “Appendix 2: Search Strategies” | 8 |
| **7** | Study screening methods | The titles and abstracts of retrieved citations were scanned by three reviewers. Full papers were accessed for all potentially relevant abstracts. Full papers were reviewed by one reviewer and were included if they met the inclusion criteria | 8 |
| **8** | Study characteristics | The characteristics of the included studies are presented in Appendix 1. | Appendices page 11-15 |
| **9** | Study selection results | The studies screened are described in Figure 1 (flow diagram) | 12 |
| **10** | Rationale for appraisal | One reviewer formally assessed quality of findings | 9 |
| **11** | Appraisal items | The CASP tool was used to appraise all included studies | 9 |
| **12** | Appraisal process | Quality assessment was conducted by one reviewer | 9 |
| **13** | Appraisal results | Study appraisal results are included in Appendix 3: Characteristics of Included Studies. | Appendices page 11-15 |
| **14** | Data extraction | One reviewer conducted data extraction using an adapted template. All text under Methods and Results were considered data from the primary studies. Data was extracted on an electronic template and then entered into a computer software for data management and analysis. | 9 |
| **15** | Software | NVivo 12.0 | 10 |
| **16** | Number of reviewers | Three reviewers for abstract/title screening, One reviewer for remainder of review process. | 8-9 |
| **17** | Coding | Line by line coding was conducted by one reviewer | 9-10 |
| **18** | Study comparison | 5 studies were selected initially to develop a code book. Subsequent studies were then coded using the code book, with new concepts created where necessary | 9-10 |
| **19** | Derivation of themes | Themes were derived inductively | 9 |
| **20** | Quotations | Quotations from the primary studies are provided throughout the results | 14-27 |
| **21** | Synthesis output | The key concepts demonstrate key experiences and perspectives of women in existing qualitative work, across society, and will aid in the development of interventions as well as guiding further research. | 27- 33 |

**Appendix 2: Search Strategies**

**MEDLINE: Epub Ahead of Print, In-Process & Other Non-Indexed Citations, MEDLINE Daily and MEDLINE 1946 to 20 June 2019, Ovid**

| **#** | **Searches** | **Results** |
| --- | --- | --- |
| 1 | gender-based violence/or intimate partner violence/or spouse abuse/ | 8864 |
| 2 | reproduct* coercion.mp. [mp=title, abstract, original title, name of substance word, subject heading word, floating sub-heading word, keyword heading word, organism supplementary concept word, protocol supplementary concept word, rare disease supplementary concept word, unique identifier, synonyms] | 76 |
| 3 | reproduct* control.mp. [mp=title, abstract, original title, name of substance word, subject heading word, floating sub-heading word, keyword heading word, organism supplementary concept word, protocol supplementary concept word, rare disease supplementary concept word, unique identifier, synonyms] | 1663 |
| 4 | pregnan* coerc*.mp. [mp=title, abstract, original title, name of substance word, subject heading word, floating sub-heading word, keyword heading word, organism supplementary concept word, protocol supplementary concept word, rare disease supplementary concept word, unique identifier, synonyms] | 22 |
| 5 | contracept* sabotage.mp. [mp=title, abstract, original title, name of substance word, subject heading word, floating sub-heading word, keyword heading word, organism supplementary concept word, protocol supplementary concept word, rare disease supplementary concept word, unique identifier, synonyms] | 8 |
| 6 | reproduct* abuse.mp. [mp=title, abstract, original title, name of substance word, subject heading word, floating sub-heading word, keyword heading word, organism supplementary concept word, protocol supplementary concept word, rare disease supplementary concept word, unique identifier, synonyms] | 2 |
| 7 | (reproduct* adj10 coerc*).mp. [mp=title, abstract, original title, name of substance word, subject heading word, floating sub-heading word, keyword heading word, organism supplementary concept word, protocol supplementary concept word, rare disease supplementary concept word, unique identifier, synonyms] | 160 |
| 8 | pregnan*.mp. [mp=title, abstract, original title, name of substance word, subject heading word, floating sub-heading word, keyword heading word, organism supplementary concept word, protocol supplementary concept word, rare disease supplementary concept word, unique identifier, synonyms] | 965263 |
| 9 | reproduct*.mp. [mp=title, abstract, original title, name of substance word, subject heading word, floating sub-heading word, keyword heading word, organism supplementary concept word, protocol supplementary concept word, rare disease supplementary concept word, unique identifier, synonyms] | 278117 |
| 10 | intimate partner violence.mp. [mp=title, abstract, original title, name of substance word, subject heading word, floating sub-heading word, keyword heading word, organism supplementary concept word, protocol supplementary concept word, rare disease supplementary concept word, unique identifier, synonyms] | 7288 |
| 11 | gender-based violence.mp. [mp=title, abstract, original title, name of substance word, subject heading word, floating sub-heading word, keyword heading word, organism supplementary concept word, protocol supplementary concept word, rare disease supplementary concept word, unique identifier, synonyms] | 831 |
| 12 | spouse abuse.mp. [mp=title, abstract, original title, name of substance word, subject heading word, floating sub-heading word, keyword heading word, organism supplementary concept word, protocol supplementary concept word, rare disease supplementary concept word, unique identifier, synonyms] | 7348 |
| 13 | domestic violence.mp. [mp=title, abstract, original title, name of substance word, subject heading word, floating sub-heading word, keyword heading word, organism supplementary concept word, protocol supplementary concept word, rare disease supplementary concept word, unique identifier, synonyms] | 9872 |
| 14 | 1 or 2 or 3 or 4 or 5 or 6 or 7 | 10643 |
| 15 | 8 or 9 | 1171821 |
| 16 | 10 or 11 or 12 or 13 | 18968 |
| 17 | 15 and 16 | 2866 |
| 18 | 14 or 17 | 12066 |
| 19 | Limit 18 to “qualitative (best balance of sensitivity and specificity)” | **4448** |

**Embase Ovid**

**Embase classic + Embase 1947 to 20 June 2019**

| **#** | **Searches** | **Results** |
| --- | --- | --- |
| 1 | gender-based violence/or intimate partner violence/or spouse abuse/ | 11572 |
| 2 | reproduct* coercion.mp. [mp=title, abstract, heading word, drug trade name, original title, device manufacturer, drug manufacturer, device trade name, keyword, floating subheading word, candidate term word] | 88 |
| 3 | reproduct* control.mp. [mp=title, abstract, heading word, drug trade name, original title, device manufacturer, drug manufacturer, device trade name, keyword, floating subheading word, candidate term word] | 259 |
| 4 | pregnan* coerc*.mp. [mp=title, abstract, heading word, drug trade name, original title, device manufacturer, drug manufacturer, device trade name, keyword, floating subheading word, candidate term word] | 28 |
| 5 | contracept* sabotage.mp. [mp=title, abstract, heading word, drug trade name, original title, device manufacturer, drug manufacturer, device trade name, keyword, floating subheading word, candidate term word] | 10 |
| 6 | reproduct* abuse.mp. [mp=title, abstract, heading word, drug trade name, original title, device manufacturer, drug manufacturer, device trade name, keyword, floating subheading word, candidate term word] | 1 |
| 7 | (reproduct* adj50 coerc*).mp. [mp=title, abstract, heading word, drug trade name, original title, device manufacturer, drug manufacturer, device trade name, keyword, floating subheading word, candidate term word] | 191 |
| 8 | pregnan*.mp. [mp=title, abstract, heading word, drug trade name, original title, device manufacturer, drug manufacturer, device trade name, keyword, floating subheading word, candidate term word] | 1044554 |
| 9 | reproduct*.mp. [mp=title, abstract, heading word, drug trade name, original title, device manufacturer, drug manufacturer, device trade name, keyword, floating subheading word, candidate term word] | 354174 |
| 10 | intimate partner violence.mp. [mp=title, abstract, heading word, drug trade name, original title, device manufacturer, drug manufacturer, device trade name, keyword, floating subheading word, candidate term word] | 7271 |
| 11 | gender-based violence.mp. [mp=title, abstract, heading word, drug trade name, original title, device manufacturer, drug manufacturer, device trade name, keyword, floating subheading word, candidate term word] | 1110 |
| 12 | spouse abuse.mp. [mp=title, abstract, heading word, drug trade name, original title, device manufacturer, drug manufacturer, device trade name, keyword, floating subheading word, candidate term word] | 419 |
| 13 | domestic violence.mp. [mp=title, abstract, heading word, drug trade name, original title, device manufacturer, drug manufacturer, device trade name, keyword, floating subheading word, candidate term word] | 11945 |
| 14 | 1 or 2 or 3 or 4 or 5 or 6 or 7 | 11937 |
| 15 | 8 or 9 | 1307338 |
| 16 | 10 or 11 or 12 or 13 | 18180 |
| 17 | 15 and 16 | 3004 |
| 18 | 14 or 17 | 13429 |
| 19 | Limit 18 to “qualitative (best balance of sensitivity and specificity)” | **4543** |

**Cinahl, EbscoHost**

**Cinahl 1981 to 20 June 2019, EbscoHost**

| **#** | **Query** | **Results** |
| --- | --- | --- |
| S19 | S14 OR S17 (Limiters – **Clinical Queries: Qualitative – Best Balance**) | **1,450** |
| S18 | S14 OR S17 | 16,707 |
| S17 | S15 AND S16 | 1,194 |
| S16 | S10 OR S11 OR S12 OR S13 | 10,655 |
| S15 | S8 OR S9 | 128,447 |
| S14 | S1 OR S2 OR S3 OR S4 OR S5 OR S6 OR S7 | 16,342 |
| S13 | TI domestic violence OR AB domestic violence | 5,072 |
| S12 | TI spouse abuse OR AB spouse abuse | 141 |
| S11 | TI gender based violence OR AB gender based violence | 484 |
| S10 | TI intimate partner violence OR AB intimate partner violence | 5,615 |
| S9 | TI reproduct* OR AB reproduct* | 25,249 |
| S8 | TI pregnan* OR AB pregnan* | 110,400 |
| S7 | TI reproduct* N10 coerc* OR AB reproduct* N10 coerc* | 102 |
| S6 | TI reproduct* abuse OR AB reproduct* abuse | 54 |
| S5 | TI contracept* sabotage OR AB contracept* sabotage | 6 |
| S4 | TI pregnan* coerc* OR AB pregnan* coerc* | 47 |
| S3 | TI reproduct* control OR AB reproduct* control | 321 |
| S2 | TI reproduct* coercion OR AB reproduct* coercion | 83 |
| S1 | (MH "Intimate Partner Violence") OR (MH "Domestic Violence") OR (MH "Gender-Based Violence") | 15,923 |

**Appendix 3: Critical appraisal of included studies with all columns from the CASP tool described, EXCEPT for overall assessment**

| **Authors** | **Was there a statement of the aims of the research? (YES, NO, PARTIAL, UNCLEAR)** | **Given the aim of the study, was a qualitative methodology appropriate? (YES, NO, PARTIAL, UNCLEAR)** | **Was the research design appropriate to address the aims of the research? (YES, NO, PARTIAL, UNCLEAR)** | **Was the recruitment strategy appropriate to the aims of the research? (YES, NO, PARTIAL, UNCLEAR)** | **Was the relationship between the researcher and participants adequately considered? (YES, NO, PARTIAL, UNCLEAR)** | **Have ethical issues been taken into consideration? (YES, NO, PARTIAL, UNCLEAR)** | **Was the data analysis sufficiently rigorous? (YES, NO, PARTIAL, UNCLEAR)** | **Were the findings supported by the evidence? (YES/NO/PARTIAL/UNCLEAR)** | **How valuable is the research?** |
| --- | --- | --- | --- | --- | --- | --- | --- | --- | --- |
| **Alhusen 2019** | Yes | Yes | Yes | Yes | No | Yes | Yes | Yes | very valuable |
| **Bagwell-Grey 2019** | Yes (clearly stated pg 6) | Yes (womens experiences of sexual violence in iitimate partner relationships) | Yes | Yes (purposive sampling at DV agency/out-patient counselling program) | Partial (experienced researcher but no reflexivity) | Yes (ethical approval and informed consent) | Yes | Yes | Very Valuable |
| **Baird 2016** | Yes (clearly stated in pg 2401) | Yes (interest in exploring intentions and experiences) | Yes (IDI) | Yes (purposive sampling at women's support agencies/refuges through posters) | Partial (brief professional description of data section but no reflexity section) | Yes (ethical approval and informed consent) | Yes (extensive explanation of experiential analysis) | Yes | Very Valuable |
| **Barber 2018** | yes | yes | yes | yes | no | no | unclear | yes | valuable |
| **Batista 2020** | Yes | Yes | Yes | Yes | No | Yes | Partial | Yes | valuable |
| **Borrero 2008** | Yes (clearly stated in pg 151) | Partial (aim to explore relationship between pregnancy intention and contraceptive use, quantitative methods could also have been used as author presented some results in quantitative form (i.e. 44% of participants)) | Yes (Semistructured interviews | Yes (purposive sampling at 7 repro health clinics) | Partial (mentions researcher experienced in diverse pop but no reflexivity section | Partial (Ethical approval but no consent specified - discusses participants names being omitted) | Yes (content analysis) | Yes | Very Valuable |
| **Boyce 2020** | Yes | Yes | Yes | Yes | Partial | Yes | Yes | Yes | very valuable |
| **Campbell 1995** | Yes (clearly stated pg 214) | Yes (intentions) | Yes | Yes (purposive sampling from womens shelter) | No (no reflexive statements) | Partial (Consent but no ethical approval mentioned) | Unclear (very brief description of thematic analysis used) | Yes | Valuable |
| **Coggins 2003** | Partial | Unclear (relationships?) | unclear of aims | partial (convenience sampling) | No (no reflexive statements) | yes (ethical approval and informed consent) | yes | partial (made big assumptions) | somewhat valuable (made big assumptions) |
| **Dasari 2016** | yes (pg 104) | yes (interested in womens experiences) | yes | yes | no (no reflexive statements) | partial (only consent no ethical approval) | yes | yes | Very Valuable |
| **Douglas 2011** | Yes (pg 341) | partial (interested in relationship) | yes | yes | no | Partial (pseudonyms for confidentiality - no info on consent or ethical approval) | unclear - no real description (could be on other study?) | yes - quotes - no major assumptions | somewhat valuable |
| **Edin 2013** | yes (pg 2) | yes (interest in womens narratives) | yes (concerns about recruitment as carried out by coordinators of the shelter (could be issues with extra inclusion/exclusion criteria)) | Yes (purpsive sampling) | No (no reflexive statements) | partial (Ethical approval but no consent specified - but WHO ethical research standards followed) | yes | yes | Very Valuable |
| **Feld 2019** | Yes | Yes | yes | yes | partial | yes | yes | yes | very valuable |
| **Grace 2020** | Yes | Yes | Yes | Yes | Partial | Yes | Yes | Yes | very valuable |
| **Griffiths 2013** | yes (pg 1) | yes (pregnancy intention) | yes | yes (purposive sampling) | yes | yes | yes | yes | Very Valuable |
| **Hathaway 2005** | yes (pg 42) | yes (interested in womens repro choices) | yes | partial (convenience sampling) | partial (descrbes interviewer as bilingual and latina and appropriate for the study but no reflexive statements) | yes | yes | yes | Very Valuable |
| **Holliday 2018** | yes (cleary stated pg 205) | yes (womens narratives) | yes | Yes (purposive sampling) | no | partial (Ethical approval but no consent specified (participants may have already consented in RCT)) | yes | yes | Very Valuable |
| **Levesque 2019** | yes (pg 6-7) | yes (womens acknowledgement and percetptoons) | yes | yes | no | yes | yes | yes | Very valuable |
| **Miller 2007** | yes (pg 360) | yes (social context) | yes | yes | partial (description of interviewers female trained in the subject) | yes | yes | yes | very valuable |
| **Mitchell 2020** | Yes | Yes | Yes | Yes | No | Yes | Partial | Yes | valuable |
| **Moore 2010** | partial (defining the different types of reproductive control perpetrated by men, examining the behaviors along a temporal continuum.) | partial (could have done quant) | yes | yes | no | yes | yes | yes | valuable |
| **Nayebi 2019** | Yes | Partial | Unclear | Yes | No | Yes | Yes | Yes | somewhat valuable |
| **Nikolajski 2015** | yes (pg 216) | yes | yes | yes | no | partial (ethical approval no consent | yes | yes | Very valuable |
| **O’Connor-Terry 2020** | Yes | Yes | Yes | Yes | Partial | Yes | Yes | Yes | very valuable |
| **Obare 2020** | Yes | Yes | Yes | Yes | Partial | Yes | Partial | Yes | very valuable |
| **Ontiri 2021** | Yes | Yes | Yes | Yes | Partial | Yes | Yes | Yes | very valuable |
| **Paterno 2018** | yes | yes | yes | yes | no | yes | yes | yes | very valuable |
| **Paul 2015** | yes (pg 312) | yes (womens decisions, social context) | yes | yes | Partial (pg 314 - had field diary with reflections - A field diary with reflections and experiences was kept and used in the interpretation and contextualization of data) | yes | yes | yes | Very Valuable |
| **Puri 2011** | yes (pg 117) | yes | yes | yes | No (no reflexive statements) | yes | yes | yes | Very Valuable |
| **Tarzia 2020** | Yes | Yes | Yes | Yes | No | Yes | Partial | Yes | Valuable |
| **Uysal 2020** | Yes | Yes | Yes | Yes | Partial | Yes | Yes | Yes | very valuable |
| **Wilson-Williams 2008** | yes (pg 1118) | partial (discusses perceptions of domestic violence and relationship with contraception - potentially quant element would be more effective for relationship) | no - should have done IDI rather than FGD due to shy participants | yes (purposive sampling) | no | No (Meeting of community consent conducted but no formal consent or ethical approval mentioned | partial - too brief | Yes | somewhat valuable |
| **Wood 2020** | Yes | Yes | yes | yes | partial | yes | yes | yes | very valuable |

**Appendix 4: Characteristics of included studies *[ordered by author surname]***

| **Authors** | **Setting** | **Aim** | **Data collection & analysis method** | **Type of RC behaviour** | **Population** |
| --- | --- | --- | --- | --- | --- |
| **Alhusen 2019** | USA | To explore associations between experiences of reproductive coercion and UIP among women with disabilities. | Semi-structured interviews; Content analysis | Pregnany coercion, sexual violence | Women aged 19 to 44, who reported diverse disabilities. Over 50% were non-hispanic white, and majority reported being unemployed. |
| **Bagwell-Grey 2019** | Southwest, USA | To describe how women describe experience of sexual violence in intimate partner relationships | Semi-structured interviews; Thematic analysis | Pregnancy coercion, contraceptive control | Women >18 years who experienced at least one type of IPV in their lifetime |
| **Baird 2016** | South West England, UK | To explore women’s pregnancy intentions and their experiences of IPV across the perinatal period | In-depth interviews; Experiential data analysis | Pregnancy coercion, contraceptive control, control of pregnancy outcome | Women 18 years and older, currently pregnant or had been pregnant within the last two years, separated from their partner at the time of the interviews and accessing support services. |
| **Barber 2018** | Michigan County, USA | To investigate the extent to which reproductive coercion accounts for any links between IPV and subsequent pregnancy | Semi-structured interviews; Thematic analysis | Pregnancy continuation, pregnancy pressure, entrapment of women, contraceptive sabotage, condom refusal | Women aged 18-22, African American and Caucasian, 10 interviews conducted with each group (poor white, poor non-white, non-poor white, non-poor non-white) |
| **Batista 2020** | Urban, Brazil | To describe the perceptions of women, prisoners or partners of imprisoned men, who experience/experienced intimate partner violence, and how they cope. | Semi-structured interviews; Content analysis and thematic modality | Forced termination | Women were between 21 and 58 years old. Most of them were white, had incomplete elementary schooling and had on average two children. Regarding marital status, 11 were cohabiting or married, and 10 were separated from their aggressor partners. |
| **Borrero 2008** | Urban Pittsburgh, USA | To better typologise pregnancy intention, understand the relationship between pregnancy intention and contraceptive use, and identify the contextual factors that shape pregnancy intention and contraceptive behaviour in a population at high risk of unintended pregnancy | Semi-structured interviews; Content analysis | Contraceptive control, pregnancy coercion | Women between 18 and 45, self-identified as either African American or white, had an abortion within the prior 2 weeks, or were not pregnant but had been sexually active with a man in the previous 12 months. Low socio-economic status |
| **Boyce 2020** | Urban, Kenya | To qualitatively describe women’s and girls’ experiences of RC in Nairobi, Kenya and opportunities for clinical intervention. | Focus group discussions and in-depth interviews; Thematic content analysis | Pregnancy coercion, Familial reproductive coercion, preference for sons | Women aged 15–49 years, currently seeking family planning services. All but one interview participant was married, with two reporting that they were in a polygamous marriage (i.e., had a co-wife), and all had children (range: 1–4) |
| **Campbell 1995** | Urban, USA | To provide preliminary background information on the relationship of abuse to pregnancy intention and pregnancy resolution (abortion, adoption, unwanted infant, wanted infant) and the decision-making process leading to the pregnancy and its resolution, using focus group data | Focus group discussions; Thematic analysis | Pregnancy coercion, contraceptive control, control of pregnancy outcome | Women from wife abuse shelters, majority were poor and of mixed race and ethnicity |
| **Coggins 2003** | Urban, Midwestern, USA | To explore the relationship between domestic violence, sexual coercion, and pregnancy, and explore the reality these women face and the thought processes they employ to survive and remain in their relationship | Focus group discussions; Thematic analysis | Pregnancy coercion, contraceptive control, control of pregnancy outcome | Women who were participating in an outreach support group for survivors living in the community and women living in a women’s shelter, low socio-economic status |
| **Dasari 2016** | Pittsburgh, USA | To identify barriers to long-acting reversible contraception (LARC) uptake among homeless young women. This includes women’s perceived barriers to contraceptive use, including knowledge and access barriers and interactions with the health care system around reproductive health | Semi-structured interviews; Content analysis | Contraceptive control | Women between 18 and 24 years of age with a past year history of homelessness |
| **Douglas 2011** | Urban, Brisbane, Australia | To understand better how reproductive coercion and sexual abuse are interrelated and how they manifest within violent relationships | In-depth interviews; Thematic analysis | Contraceptive control, pregnancy coercion | Women were all over 18 years old, had in the past six months leading up to the first interview experienced DFV (Domestic and Family Violence) from their current or previous intimate partner and engaged with the legal system in some way to respond to the violence. |
| **Edin 2013** | Sweden | To describe and analyse nine Swedish women’s retrospective stories about IPV with a focus on power and coping strategies as intimate partners, particularly regarding experiences of sex, contraception, and becoming pregnant. | Semi-structured interviews; Narrative analysis | Pregnancy coercion, contraceptive control | Women were approximately between 31 and 55 years old and had been subjected to violence by an intimate partner in a relationship in which they had become pregnant. |
| **Feld 2019** | Peri-urban, Santo Domingo de los Tsáchilas, Ecuador | To describe the structural, social, economic context of pregnancy intention in a peri-urban, diverse, low-resource community in Ecuador. | In-depth semi-structured interviews; Content analysis | Contraceptive control | Nine of the participants self-identified as Mestizo and 10 represented a minority race or ethnicity in Ecuador. All of the women spoke Spanish as their first language. Most of the women did not finish high school and nine had elementary education or less. Most women were in a relationship with a man. 13 reported being unemployed and many reported informal or inconsistent work. All but one woman reported having at least one unplanned pregnancy. |
| **Grace 2020** | Urban, USA | To describe and understand the context of RC and the use of RC safety strategies among Latina women receiving services at an urban clinic, through listening to the experiences of the women in their own words. | Semi-structured interviews; Thematic analysis | Contraceptive control, sexual violence, coerced termination | Latina women aged 20 to 40 years who were either born in the United States (n = 2) or emigrated from Mexico (n = 2), El Salvador (n = 6), Guatemala (n = 2), or Honduras (n = 1) |
| **Griffiths 2013** | Remote, Western Desert region, Western Australia | To explore the formation and expression of pregnancy intentions in an Aboriginal population to inform health service improvements | Semi-structured interviews; Content analysis | Pregnancy coercion, contraceptive control | Remote-dwelling Aboriginal or Torres Strait Islander women aged 18-50 years. |
| **Hathaway 2005** | Massachusetts General Hospital in Boston, USA | To increase our understanding of how intimate partner abuse may limit women’s reproductive choices | Structured interviews; Content analysis | Pregnancy coercion, contraceptive control, control of pregnancy outcome | Women who were participating in a hospital-based domestic violence program. |
| **Holliday 2018** | Pittsburgh, Pennsylvania, USA | To qualitatively describe and compare contexts for unintended pregnancy risk between low-income Black and White women with histories of IPV/RC | Semi-structured interviews; Thematic analysis | Pregnancy coercion, contraceptive control | Women aged 18 to 29, African American and White women, Low-income, from family planning clinics in Western Pennsylvania, all with histories of IPV |
| **Levesque 2019** | Quebec, Canada | To explore young women’s acknowledgement of RC and the associated issues. | Individual interview/questionnaire; Thematic analysis | Contraception sabotage, pregnancy coercion, control of pregnancy outcome | Women 18-29 years, who have experienced RC in past 2 years. Majority Caucasian, minority Caribbean, North African, Asian origins |
| **Miller 2007** | USA | To examine the context of pregnancy and sexual health among adolescent females with a history of intimate partner violence (IPV). This paper reports on a subset of females who described abusive male partners’ explicit pregnancy-promoting behaviours (i.e. messages and behaviours that led females to believe their partner was actively trying to impregnate them). | Interviews; Content analysis | Pregnancy coercion, Contraceptive control | Females aged 14 to 20 years with a history of 1 or more abusive relationships (with known history of IPV, about violence, sexual experiences, and related behaviours) |
| **Mitchell 2020** | Suva, Fiji | To examine young iTaukei (Indigenous Fijian) women’s experiences of, and responses to, nonphysical forms of coercion in romantic relationships. | In-depth interviews; Inductive thematic analysis | Coerced termination | Young unmarried iTaukei women attending university in Suva, Fiji aged 18 to 26 years. |
| **Moore 2010** | Urban, one Midwest and two East Coast sites; USA | To define the different types of reproductive control perpetrated by men, examining the behaviours on a temporal continuum (before sexual intercourse, during sexual intercourse, and post-conception). | Semi-structured interviews; Thematic analysis | Contraceptive control, Pregnancy coercion, Control of pregnancy outcome | Women with a history of IPV age 18 to 49, from 3 sites; domestic violence shelter, freestanding abortion clinic, family planning clinic. |
| **Nayebi 2019** | Urban, Iran | To explain the reproductive health and rights of abused women in Tehran, Iran | Unstructured individual interviews; Content analysis | Familial reproductive coercion | 18 female victims of domestic violence, who were aged between 23 and 45 years old |
| **Nikolajski 2015** | Western Pennsylvania, USA | To explore women’s experiences with contraception sabotage and pregnancy-promoting behaviours by male partners and how may these vary by race. | Semi-structured interviews; Content analysis | Contraceptive control, Pregnancy coercion, Control of pregnancy outcome | Low income, Women aged 18 to 45, self-identified as either African American or White, currently pregnant, had an abortion within prior 2 weeks or had been sexually active with a man in previous 12 months. Recruited from reproductive health clinics. |
| **O’Connor-Terry 2020** | Urban, USA | To explore reproductive health care in situations of IPV and elucidate the ways that restrictions on Title X would affect survivors of IPV | Semi-structured interviews; Thematic analysis | Contraceptive control, coerced termination, familial reproductive coercion | Women with a mean age of 40 years old. The majority of our participants self- identified their race as Black, graduated from high school or participated in community college or tech school, and had a yearly household income below $10,000 per year. |
| **Obare 2020** | Rural, Kenya | To understand contraceptive use and provision practices that were likely to influence method discontinuation in the study setting | In-depth interviews; Inductive content analysis | Covert contraception as a form of resistance | 52% of women were aged between 20 and 29 years Fifteen participants had secondary or higher levels of education, another 15 had complete primary level education, while the rest had no formal schooling or had incomplete primary level education. Nineteen partici- pants had two or three living children at the time of recruitment, 15 had four or more children while 8 had only one living child. |
| **Ontiri 2021** | Rural, Kenya | To explore the experience with contraceptive use and discontinuation among discontinuers | Focus group discussions and in-depth interviews; Thematic analysis | Familial reproductive coercion | The majority of participants were adolescents and youth aged 15–24 years (51%), had primary education (53%), were farmers (32%), and had one to two children |
| **Paterno 2018** | Baltimore, Maryland, USA | To examine and integrate data related to experiences of reproductive coercion and its association with characteristics of intimate relationships. To address these important gaps and expand the utility of the concept of reproductive coercion in a clinical setting, we explored experiences of reproductive coercion within a romantic and sexual relationship context | In-depth interviews; Qualitative descriptive analysis | Contraceptive Control, Pregnancy coercion, Familial reproductive coercion | Young adult, low-income, primarily African American women |
| **Paul 2015** | Rural Udaipur, Rajasthan, India | To explore how young rural Indian women make reproductive decisions and negotiate childbearing and reproductive agency; under what circumstances are abortion and contraception, what are the contextually accepted means of family planning and Which contraceptive methods are available to young people, given the new and enabling policy environment and the wider range of methods in the market. | In-depth interviews; Thematic analysis | Contraceptive sabotage, Pregnancy coercion, Control of pregnancy outcome | Married (preferably recently) young women aged 18 to 24 |
| **Puri 2011** | California, New York & New Jersey, USA | To investigate and document the experiences of son preference and sex selection among Indian women who have migrated to the United States. | Semi-structured in-depth interviews; Thematic analysis | Pregnancy coercion in context of pressure to have sons, control of pregnancy outcome – termination of female foetuses. | Women who migrated from Indian subcontinent after age 18, and who have a history of seeking sex selection services. |
| **Tarzia 2020** | Urban, Australia | To understand and differentiate between women’s experiences of “stealthing” (non-consensual condom removal) and reproductive coercion and abuse (RCA) which is defined as any deliberate attempt to control a woman’s reproductive choices or inter- fere with her reproductive autonomy. | In-depth interviews; Thematic narrative analysis | Sexual violence, forced termination, familial reproductive coercion | Women were aged between 18 and 44 years, and predominantly spoke English as a first language. The majority were born in Australia and had completed some higher education. Although most were engaged in either full or part-time work, eight of the women’s main source of income was a pension or benefit. |
| **Uysal 2020** | Urban, Mexico | To qualitatively describe adolescent girls’ perceptions and experiences of RC and IPV, and their impact on girls’ reproductive health and coping strategies of their health-seeking behavior, in Tijuana, Mexico to inform development of interventions to improve reproductive autonomy and health of this marginalized population of adolescent girls. | In-depth interviews; Content analysis | Contraceptive control | Among the 20 adolescent girls, the median age was 17.5 years, the average level of education was 7 years (middle school), and the country of origin was Mexico (from Baja California and other southern states). All participants reported experiencing IPV (verbal, physical, and/or sexual) at least once in their lifetime with half in the past year and 60% reported ever experiencing RC. Only one participant reported currently using a modern contraceptive method (Depo-Provera) other than male condoms. All of the participants had at least one child under the age of five or were pregnant at the time of their interview |
| **Wilson-Williams 2008** | Rural, Gangadhar, Maharashtra, India | To examine the interrelationships between family planning use and perceptions of domestic violence in a small village in rural West India | Focus group discussions; Thematic analysis | Pregnancy coercion, preference for sons, contraceptive control | Three groups of younger married women (20-28 years) and four groups of older married women (28+ years), Low socio-economic status households |
| **Wood 2020** | Urban Nairobi, Kenya | To test and refine the RCS and examine the prevalence and severity of RC among reproductive age women experiencing IPV | In-depth, semi-structured interviews; Thematic analysis | Contraceptive control | Women age 18–35, in a relationship where physical or sexual IPV or fears for safety occurred in the past three months, a resident of the study settlements  with no plans to move within the next six months, and fluent in English or Kiswahili. |

**Appendix 5: Code Book**

| **Code Book** | **Examples** |
| --- | --- |
| **Manifestations of Reproductive Coercion** | *Specific behaviours that constitute reproductive coercion* |
| 1. **Contraceptive control** |  |
| - 1. **Contraceptive refusal** | *Condom refusal, or refusal to allow oral contraceptive pill (OCP)* |
| - 1. **Contraceptive sabotage** | *Overt contraceptive control such as throwing away birth control pills, pulling contraception (nuvaring or IUD) out of woman, biting holes in condom, etc* |
| - 1. **Contraceptive deception** | *Non-consensual condom removal, lying about using condom, dissuading woman from using contraceptive pill by exaggerating side effects or emotional coercion such as accusing woman of cheating if she continues with OCP, etc* |
|  |  |
| 1. **Pregnancy pressure/coercion** |  |
| - 1. **Emotional coercion** | *Verbal and emotional pressure such as talking about how badly partner wants a baby, threatening to leave if she doesn’t get pregnant, if woman advises she isn’t ready partner says “don’t you want to be a part of me, don’t you want to be a part of me forever?”* |
| - 1. **Physical coercion** | *Forced sex or rape with intention to get woman pregnant, or forced sex with indifference to whether woman is protected from pregnancy* |
| - 1. **Failure to conceive** | *Violence or harassment from partner if woman does not conceive* |
|  |  |
| 1. **Control of pregnancy outcome** |  |
| - 1. **Termination** | *Partner threatening violence to cause miscarriage, threatening to leave unless woman has abortion, demanding/forcing woman has abortion* |
| - 1. **Pregnancy continuation** | *Tactics such as:*  *Controlling woman’s movements to stop her having a termination*  *Begging*  *Badgering “you can’t kill my baby”*  *Making promises to support baby*  *Making woman feel guilty*  *Threats “if you kill my baby, I will kill you”* |
|  |  |
| **Reasons for Reproductive Coercion** |  |
|  |  |
| 1. **Reasons for reproductive coercion** |  |
| - 1. **To trap woman** | *Partner spoke about wanting to impregnate her to “tie her to him forever”* |
| - 1. **Wanting a child (Ego?)** | *Taking pride in being a father, believing it will make him stronger, happier* |
| - 1. **Incarceration** | *Partner monitored ovulatory cycles and sabotaged contraception efforts before incarceration*  *Pregnancy to maintain emotional and economic security while in prison*  *Impending incarceration meant partner wanted pregnancy to lesson chances of woman leaving him as she would be seen as less desirable and would invest more in relationship* |
| - 1. **Role of women** | Gender norms to bear children, not wanting to disobey or fail to fulfil husbands expectations in terms of child bearing, husbands emphasised need to produce children, strict gender roles and expectations on women around sexuality/fertility |
|  |  |
| **Effect of reproductive coercion on women?** |  |
|  |  |
| 1. **Consequences of reproductive coercion** | *Fear of STIs, psychological effects, unintended pregnancy* |
| 1. **Reasons for women’s compliance** |  |
| - 1. **Fear of abandonment** | *Love for partner eclipsed RC behaviour, fear that partner would end relationship* |
| - 1. **Dependence on partner** | *e.g. Financial dependence on partner* |
| - 1. **Fear of partner/violence** | *Fear that partner will hurt her if she disobeys wishes* |
| 1. **Women’s responses to reproductive coercion** |  |
| - 1. **Reaction – Minimising** | *Downplaying incident, “it started off as a bit of a joke when he removed the condom half way through”*  *manipulation led women to doubt seriousness of incident* |
| - 1. **Reaction – Shame** | *Women felt shame and internal conflict for disobeying husband and taking contraception in secret, women doubted their right to confront partner, difficulty self-identifying as victim* |
| - 1. **Reaction – Blaming self** | *Assuming responsibility, i.e. drinking at time of incident clouding recollection “maybe I said he could take off the condom”* |
| - 1. **Reaction – Anger** | *Woman mad that she took precautions and man took off condom like ‘he had the right to decide for her’* |
| - 1. **Women’s awareness of reproductive coercion** | *Awareness and acknowledgement depends on type of violence perpetrated*  *Factors facilitating awareness include:*  *Acquiring knowledge about the issue*  *Having a friend describe RC behaviour*  *Finding a new partner that respects contra choices* |
| - 1. **Barriers to awareness** | *Non-acknowledgement due to:*  *Assuming responsibility*  *Downplaying incident*  *Being in violent relationship*  *Difficulty identifying as victim* |
| - 1. **Help seeking** | *Ways in which women sought help* |
|  |  |
| **Resistance to Reproductive Coercion** |  |
|  |  |
| 1. **Forms of resistance** |  |
| - 1. **Fighting back** | *Women pushed off partner after realising there was no condom* |
| - 1. **Ending relationship** | *Partner coercion allowed woman to recognise controlling nature of relationship and end it* |
| - 1. **Covert contraception** | *Using contraception without partners knowledge i.e. secret depo injections, telling partner OCP are vitamins* |
| 1. **Partner’s reaction to resistance** | *Men interpreted women’s protests as emotional rejection*  *Physical and verbal violence towards woman if partner finds out she is taking covert contraception* |
| **Societal manifestations** |  |
|  |  |
| 1. **Reproductive coercion by in-laws** | *(in particular populations) Pregnancy pressure by in-laws, pressure for more children, mother-in-law influences husbands contraceptive decisions, source of pressure to have son* |
| 1. **Sex selection/Preference for sons** | *(in particular populations) Pressure for sons, clear expectation to have male child, termination of female fetus* |
| 1. **Differences in experiences across populations** | *Experiences of African American women as opposed to Caucasian, migrant women in Canada, etc* |
| 1. **Motherhood vs career?** | *Women torn between motherhood and their careers/schooling and influence in pregnancy intention (not sure if relevant here or even relevant at results level)* |
