# Supplementary material for: Women’s perceptions and experiences of reproductive coercion and abuse: a qualitative evidence synthesis
Source: PLoS One. 2021 Dec 21;16(12):e0261551. doi: 10.1371/journal.pone.0261551 (PMC8691598; doi:10.1371/journal.pone.0261551)
Supplement: Attachment — Submitted filename: Response to reviewers - RC QES_FINAL.docx [file pone.0261551.s008.docx]

Dear Editors,

Thank you and the reviewers for your thoughtful consideration of our manuscript. We have revised based on the feedback in a tracked changes version of the manuscript and explained the revisions and responded to questions in the table below, point-by-point.

Thank you for your consideration, we look forward to hearing from you.

Sincerely,

Jessica E Moulton, on behalf of the authors

| **Reviewer 1** | | | |
| --- | --- | --- | --- |
| **No** | **Section** | **Comments** | **Authors Response** |
| 1 | Overall | My main concern is that an exhaustive and comprehensive review such as this is better suited for an academic paper (as it states it was originally written) than a manuscript. I think it can be scaled back and more narrowly focused to be more appropriate for publication. Findings need to be further synthesized for readability and to avoid presenting 15 pages of data. Scope could be narrowed to focus on women’s experiences OR women’s reactions OR women’s perceptions of reasons for RC. | Thank you for your valuable feedback. We have now reframed and recategorized the findings to avoid duplication and improve readability. We hope that the findings are now clearer and more concise. Please see revisions throughout the results section and table 1. |
| 2 | Overall | The theoretical framework (section 2.1) (could be scaled back) | Thank you kindly for your comment. We have substantially reduced this section and moved to the background:  “Reproductive coercion and abuse can be explained in a social structure where cultural and social norms of male dominance over women are widely upheld (13). Men’s assertion of power and control of women through abusive tactics has been researched extensively. Heise’s highly influential ecological framework can be used for understanding the causes of gender-based violence and to conceptualise reproductive coercion and abuse as an interplay between personal, situational and sociocultural factors(13). Factors that may influence the likelihood of perpetration of reproductive coercion and abuse include personal factors such as perpetrators having experienced abuse themselves or history of abusive behaviour, interpersonal factors such as friends or family members of the perpetrator who are supportive of violence and situational and sociocultural factors such as high rates of unemployment, religion, and policy and social and cultural norms in which violence against women is permissible (13)” |
| 3 | Overall | Methods sections on lines 254-295 can be significantly scaled back. | Thank you for your comment. We have made some minor changes as per tracked changes, however feel that we are unable to scale back any further due to the requirements for the Qualitative Evidence Synthesis methodology and ENTREQ statement for enhancing transparency in reporting the synthesis of qualitative research we have provided a comprehensive methodology for the paper. |
| 4 | Overall | The Table is helpful but could be more helpful – needs to summarize topics more broadly and succinctly to be useful as a table. | Thank you for this comment. We have now refined our findings and Table 1: Summary of qualitative findings – please see revisions throughout. The Table is a requirement of the Qualitative Evidence Synthesis Methodology to report on the confidence in each finding |
| 5 | Overall | I am also wondering if the study was limited to the actual qualitative data that was presented in each study? In my study, which was included in the review, I sometimes describe a range of behaviors that emerged in interviews, and then present a quote to illustrate the theme, but my study was only cited in this review regarding the behavior in the one quote. That seems to be a limitation that should be acknowledged and more intentionally addressed. | Thank you for this really important feedback. The findings are based on each study authors interpretation of themes present in their results, including any quotes. Only data that describes women’s experiences and perceptions of reproductive coercion was included in this review, and for the behaviours related to reproductive coercion and abuse, only those where there was reproductive intent. For example, condom refusal or non-consensual removal for sexual pleasure reasons was not included in the synthesis. We hope that the updated findings are a better representation of the data. |
| 6 | Line 129 | Citation 8 is also published in 2010 – would cite earlier literature here to support the point being made. | Thank you for this, we have now updated the sentence as reproduced below;  “However, well before this, pregnancy-controlling behaviours by male partners have been described in the gender-based violence literature without being identified as reproductive coercion (Grace, 2018 #64)”  We felt that some of the studies included in the Systematic Review by Grace 2018, were a better representation than Citation 3 and 8. The systematic review included studies with examples of reproductive coercion behaviours that were published prior to 2010. |
| 7 | Line 132: | Some studies find greater than 16% prevalence, for example: • Alexander, K. A., Willie, T. C., McDonald-Mosley, R., Campbell, J. C., Miller, E., & Decker, M. R. (2019). Associations Between Reproductive Coercion, Partner Violence, and Mental Health Symptoms Among Young Black Women in Baltimore, Maryland. Journal of Interpersonal Violence, 088626051986090. <https://doi.org/10.1177/0886260519860900> • Grace KT, Perrin N, Clough A, Miller E, Glass NE. Correlates of reproductive coercion among college women in abusive relationships: Baseline data from the College Safety Study. J Am Coll Heal 2020;0:1–8. <https://doi.org/10.1080/07448481.2020.1790570>. • Holliday CN, McCauley HL, Silverman JG, et al. Racial/Ethnic Differences in Women’s Experiences of Reproductive Coercion, Intimate Partner Violence, and Unintended Pregnancy. J Women’s Heal. 2017;26(8):1-8. doi:10.1089/jwh.2016.5996 | Thank you kindly for these references. We have now updated the prevalence rates as reproduced below;  “Prevalence rates of reproductive coercion and abuse have ranged from 8.6% of all women in The National Intimate Partner and Sexual Violence Survey in the United States of America (USA) (Black, 2011), to as high as one in three (37.8%) young self-identified Black or African American women in a cross-sectional study in Baltimore, Maryland (Alexander, 2021).” |
| 8 | Lines 224-227 | Seem to repeat the paragraph above? | Thank you for this comment. We have now deleted the former paragraph and the definition of our topic of interest is as follows:  “Reproductive coercion was broadly conceptualised as contraceptive sabotage, pregnancy coercion and control of pregnancy outcomes as defined in the Marie Stopes ‘Hidden Forces’ White Paper on Reproductive Coercion (Marie Stopes Australia, 2018). Behaviours that constitute reproductive coercion included 1) sabotaging another’s contraception; 2) controlling a pregnancy outcome (forcing someone to have a termination or continue a pregnancy); 3) forcing or pressuring someone into pregnancy; and 4) forcing someone to have a sterilisation procedure (Marie Stopes Australia, 2018 ).” |
| 9 | Lines 308-309 | “All women in the studies were in relationships with male partners at the time they experienced reproductive coercion.” I think it’s more accurate to say all women experienced RC from male partners. Can’t necessarily say they were in a relationship. | Thank you for this excellent comment. We agree that it is more accurate to say “all women experienced RC from male partners” and have  corrected in track changes. |
| 10 | Lines 514-515 | Are studies of men included in this review? Some studies are absent if so (Alexander, K. A., Sanders, R. A., Grace, K. T., Thorpe, R. J., Doro, E., & Bowleg, L. (2019). “Having a Child Meant I Had a Real Life”: Reproductive Coercion and Childbearing Motivations among Young Black Men Living in Baltimore. Journal of Interpersonal Violence, 1–29. <https://doi.org/10.1177/0886260519853400>) | Thank you for this comment. We only included studies of cis women’s experiences and perceptions, however, acknowledge that future research on motivations for perpetrating RC would be highly valuable. |
| 11 | Study Flow Diagram: | “reports not retrieved” – should this say “unable to be retrieved”? Or is there another reason they were not retrieved? | Thank you for your comment on this. We have now amended the study flow diagram to say “unable to be retrieved” for clarity. |
| 12 | Study Flow Diagram: | What does “wrong setting” and “wrong population” refer to? | Thank you for your comment. Wrong setting and wrong population in the context of the study flow diagram are to indicate that the articles did not meet the selection criteria for this review, for example, the 1 study excluded due to wrong population may have explored the experiences and perceptions of health care workers in treating/supporting someone who has experienced RC rather than the person themselves. The 2 studies in the “wrong setting” have been amended to “wrong study design”, with other studies that did not include a qualitative study design and analysis. |
| 13 | Study Flow Diagram: | What is the difference between the 2 categories in the “Included” section? | Thank you for noting our error – we have deleted the second category “reports” as it is duplicative. |
| 14 | Logic model | This is really helpful in synthesizing the large amount of data in this paper. Some comments: Accusations of infidelity is a tactic, not a cause of RC. I am not seeing the cultural piece among causes? Numbers need to be explained. “Women’s partners reaction to resistance” should really move one column to the right, since this is referring to a reaction to an immediate outcome. Section on “Short to Long Term Outcomes – Barriers to Services” is less clear to me as a distinct category. I think it could be eliminated altogether or else the scope of the paper could be narrowed to “Outcomes”, and then this section could be fleshed out and organized a bit more. | Thank you for your feedback on the logic model, Please see the revisions in the figure (not marked as tracked changes). We note that the cultural factors are captured in the boxes: systemic social inequalities, son preferences, family pressure, migration or visa status, gender roles. |
| 15 | Line 224 | “constitute” | Corrected; in track changes. |
| 16 | Line 402 | suggest word other than “heartbreak” | Thank you for this suggestion. We have amended “heartbreak” to “significant grief” |
| 17 | Line 468: | “experienced” | Corrected; in track changes. |
| 18 | Line 882: | suggest “may” instead of “will | Corrected; in track changes. |

| **Reviewer 2** | | | |
| --- | --- | --- | --- |
| **No** | **Section** | **Comments** | **Authors Response** |
| 1 | Overall | I would strongly suggest that the authors critically engage with the work of Tarzia and Hegarty (2021). Their conceptual work around RC would assist in both interpreting the findings of this review and in ensuring that there is clarity around the behaviours being described | Thank you kindly for your review, and for this excellent suggestion – a fantastic article.  We have now engaged with the suggested work and have made the following changes:   - In Topic of Interest, Section 2.1 \| Criteria for considering studies for this synthesis, we have added the following: “As posited in a conceptual re-evaluation of the term reproductive coercion, when exploring experiences of reproductive coercion, only behaviours where there is a perceived explicit intent of either impregnating a woman, preventing her from becoming pregnant or ending a pregnancy will be considered.” - For Finding 1 we have added the following: “While many women in the studies identified condom refusal and non-consensual removal in conjunction with other behaviours consistent with reproductive coercion and abuse, it is important to note that these forms of sexual violence can occur in isolation of intention to conceive (50). Some studies noted additional contraceptive sabotage behaviours, however as it was unclear whether the intent was to cause pregnancy, these data have not been included in this finding. We also did not consider condom refusal to be reproductive coercion and abuse when women explicitly stated it was due to sexual pleasure concerns”. |
| 2 | Terminology | Although recent research has opted for the terms "reproductive coercion and abuse" or "reproductive abuse" the authors have chosen to stick to the older term "reproductive coercion". This is absolutely fine but I would like to see a justification for why they have done this. I assume it is to avoid confusion since most of the literature they are reviewing will use RC but it would be good to state this explicitly. | Thank you for this comment. We had originally stated “In this paper reproductive coercion and abuse will herein be referred to as reproductive coercion, however this is not to diminish the severity of abuse experienced by survivors.”, however agree that recent research favours reproductive coercion and abuse. We have now amended “reproductive coercion” to “reproductive coercion and abuse” as per tracked changes. |
| 3 | Line 132 | Please note that the highest prevalence estimates are around 30%. Please see the recent systematic review by Rowland and Walker for a summary of prevalence rates across different settings. | Thank you kindly for this comment, we have now updated the prevalence rates as reproduced below;  “Prevalence rates of reproductive coercion and abuse have ranged from 8.6% of all women in The National Intimate Partner and Sexual Violence Survey in the United States of America (USA) (Black, 2011), to as high as one in three (37.8%) young self-identified black/African American women in a cross-sectional study in Baltimore, Maryland (Alexander, 2021 ).” |
| 4 | Line 141 | The authors outline some of the background literature on risk factors and prevalence. A sentence should be added here qualifying that measurement issues render some of this work problematic given that different behaviours are included across different studies and many studies are either over or under-inclusive. The Tarzia & Hegarty 2021 paper outlines this issue. | Thank you for this really important point. We have included the following sentence in the introduction to highlight the issues with current prevalence rates:  “However, a lack of consistent measurement and conceptual clarity around reproductive coercion and abuse mean that true prevalence rates are inconclusive (9).” |
| 5 | Line 149 | Should have a citation. Suggest Tarzia L, Douglas H, Sheeran N. Reproductive coercion and abuse against women from minority ethnic backgrounds: views of service providers in Australia. Cult Health Sex. 2021 Jan 11:1-28. | Thank you for this comment and reference. We have added the citation as per tracked changes. |
| 6 | Line 176 | The authors state that the review takes a phenomenological approach, however, the findings do not align with this. Simply reporting types of behaviours and women's responses to them does not describe the meanings participants place on their experiences. Not everything that examines people's experiences in a qualitative way is "phenomenological". Please reconsider whether another approach describes your analysis more accurately. | Thank you for this important comment. We agree that a phenomenological approach does not accurately describe the findings in the review, and have opted to delete the section on theoretical frameworks as the study is rather conducted in accordance to the Qualitative Evidence Synthesis Methodology with thematic analysis of data. |
| 7 | Line 181 | Similar to my previous point, the authors claim to have drawn on a number of frameworks to help them interpret the findings, without insufficient critical engagement with what these frameworks actually do. For example, acknowledging gendered power relations in society is just... feminism? Connell's gender regime (from what I 8understand) is a particular way of describing gendered structural relations within "an organisation". If you are simply talking about broader gender inequality in society then I don't think Connell's theory is really what you need. To be clear I am not disagreeing with your argument that social structures condition men's and women's social roles, just that Connell's theory is being mis-applied or not adequately explained. Similarly, the claim that the "ecological framework" has been used to interpret the findings needs further explanation or justification. If all you are doing is acknowledging the cultural specificity of women's experiences of RC then this is not "ecological". The ecological framework does a very specific thing which is look at VAW at all levels (individual, relationship, community, society) and you have not really done that in interpreting your findings here. Neither is there any evidence (contrary to the claims in Line 191 onwards) that the ecological factors identified by Heise in relation to VAW more broadly are actually applicable to the RC context. To be clear I am not suggesting you rework your findings just that you avoid using fancy frameworks to describe what you did unless they are actually being used properly. | Thank you kindly for your comment. We have substantially reduced this section and moved to the background:  “Reproductive coercion and abuse can be explained in a social structure where cultural and social norms of male dominance over women are widely upheld (13). Men’s assertion of power and control of women through abusive tactics has been researched extensively. Heise’s highly influential ecological framework can be used for understanding the causes of gender-based violence and to conceptualise reproductive coercion and abuse as an interplay between personal, situational and sociocultural factors(13). Factors that may influence the likelihood of perpetration of reproductive coercion and abuse include personal factors such as perpetrators having experienced abuse themselves or history of abusive behaviour, interpersonal factors such as friends or family members of the perpetrator who are supportive of violence and situational and sociocultural factors such as high rates of unemployment, religion, and policy and social and cultural norms in which violence against women is permissible (13)” |
| 8 | Lines 217-219 | The definition and measurement tool are problematic. Please justify why these definitions have been used as opposed to more recent definitions and frameworks (see Tarzia & Hegarty 2021). For example Miller's measurement tool completely excludes abortion coercion as a form of RC. Happily I don't think you actually *did* use the Miller measurement tool in your analysis since the rest of the paper is very specific about the intent of the behaviour so not really sure why this is here. | Thank you for this comment. We have updated the *Topic of Interest* section to solely refer to the definition as included in the Marie Stopes ‘*Hidden Forces*’ White Paper and deleted reference to the Miller measurement tool as per tracked changes. We have also highlighted the importance of intent as per Tarzia and Hegarty 2021 in this section. |
| 9 | Line 261 | Please explain briefly how the CASP was adapted | We adapted the tool to remove the overall assessment of methodological limitations. This approach is suggested by Cochrane for qualitative evidence syntheses. Instead of reporting the overall assessment, we report the specific concerns of each of the critical appraisal domains. We have revised in the text and added a table reporting the methodological limitations in an appendix: We did not conduct an overall assessment ; rather, the concerns regarding each of these domains is described in detail Appendix 3. |
| 10 | Lines 366 onwards (Results section) | Do all of the studies describing contraceptive sabotage do so in the context of DELIBERATE pregnancy intention (as opposed to "stealthing")? It is fantastic to see the specific attention paid to this detail (intent) throughout the analysis, however, it is less clear whether the included studies have been curated with this exclusion in mind? For example, the quotes on lines 387-388 clearly do not relate to pregnancy intent but rather, to other motivations for removing a condom. Please clarify. | Thank you for this feedback. In the process of reframing findings we assessed all data included in the finding and have removed any data that was not with explicit pregnancy intent. We have also added the following:  “While many women in the studies identified condom refusal and non-consensual removal in conjunction with other behaviours consistent with reproductive coercion and abuse, it is important to note that these forms of sexual violence can occur in isolation of intention to conceive (Tarzia, 2020 ). Some studies noted additional contraceptive sabotage behaviours, however as it was unclear whether the intent was to cause pregnancy, these findings have not been included in this finding (Campbell, Coggins, Paterno, Grace, O’Connor Terry, Levesque). We also did not consider condom refusal to be reproductive coercion and abuse when women explicitly stated it was due to sexual pleasure concerns.” |
| 11 | Results | There is a broader issue of overlap and confusion within the Findings. There are some Findings that do not seem to fit well under their respective theme headings, and there are many areas of overlap. I would strongly recommend the authors review the Findings with the aim of consolidating some of them and re-assessing the appropriateness of the headings. For example, Finding 10 suggests that men perpetrate RC as a way of bolstering their ego. However, it's unclear how the men "showing off the baby" necessarily demonstrates reproductive coercion? Is it clear that in these studies, the women believed that men wanted a child to satisfy their egos - AND specifically coerced them into having one? | Thank you for this comment. We have consolidated and reframed findings. We removed any Findings that were unclear about reproductive intent such as Finding 10. Please see revisions throughout the Results section and Summary of qualitative findings table. |
| 12 | Results | Similarly, Finding 13 does not seem to be describing a motivation for perpetrating RC. Finding 15 includes a lot of extra content that describes the behaviours/mechanisms of RC but is not relevant to understanding WHY the perpetrator does it (which is the focus of the theme it is situated under) | We merged Finding 13 with Finding 7, and have reframed as a “Consequence” of RC as opposed to a motivation. Similarly with Finding 15 (now Finding 13) we have amended the finding to reduce duplication of RC behaviours and reframed as a societal/cultural phenomenon. Please see revisions throughout the Results section and Summary of qualitative findings table. |
| 13 | Results | This issue of overlap is particularly problematic in the themes on Barriers. A lot of the content described under "Barriers to accessing IPV interventions" for instance is not actually about RC as a barrier to IPV interventions. The same goes for "Barriers to termination" and "Barriers to contraception". A lot of this content seems to sit better under the types of behaviours that perpetrators use. | Thank you kindly for this comment, we have now reframed the findings, and merged many of these later findings with existing ones that detail behaviours that perpetrating use within “Women’s experiences of reproductive coercion and abuse”. Please see revisions throughout the Results section and Summary of qualitative findings table. |
| 14 | Discussion | The Discussion section needs some minor revisions relating to my comments above (regarding the definitional issues and use of frameworks). | Thank you for this comment. We have made the following changes to the Discussion:   - Removed the paragraph related to the theoretical frameworks - Included this sentence in strengths of the study: “Another key strength was that we only included data describing the types of behaviours that perpetrators use when there was clear intent of a reproductive outcome, particularly pertaining to contraceptive sabotage” - Other amendments as per tracked changes |
